# High-Temperature Tensile Mechanical Properties and Microstructure of Rolled 6082-T6 Aluminum Alloy Sheets

**DOI:** 10.3390/ma16217019

**Published:** 2023-11-02

**Authors:** Tuo Ye, Erli Xia, Sawei Qiu, Yong Wang, Huanyu Yue, Jie Liu, Yuanzhi Wu

**Affiliations:** 1 School of Intelligent Manufacturing and Mechanical Engineering, Hunan Institute of Technology, Hengyang 421002, China; 2017001002@hnit.edu.cn (T.Y.); 2021001046@hnit.edu.cn (E.X.); 2022001005@hnit.edu.cn (S.Q.); 2021001023@hnit.edu.cn (Y.W.); 21010240108@xs.hnit.edu.cn (H.Y.);; 2 Research Institute of Automobile Parts Technology, Hunan Institute of Technology, Hengyang 421002, China

**Keywords:** 6082 alloy, anisotropy, mechanical properties, microstructure, texture

## Abstract

The tensile properties of rolled 6082-T6 aluminum alloy were tested at a high temperature, and the influences of tensile temperature on its flow stress and anisotropy were studied. The microstructure evolution was characterized using optical microscopy (OM), scanning electron microscopy (SEM), transmission electron microscopy (TEM) and X-ray diffraction (XRD). It was concluded that the tensile strength of the studied alloy decreases with increasing temperature. In higher-temperature deformation, the dislocation density decreases alongside the coarsening of precipitates, leading to a decrease in deformation resistance, and increase in the number of dimples and deepening of their sizes, exhibiting good plasticity. The alloy displays anisotropic mechanical properties at 200 °C due to the elongated grains and the orientation of the α-phase. The anisotropy becomes weaker with increasing temperature. There is no evident anisotropy at 400 °C, as the slip systems are activated more easily and the grains begin to recrystallize.

## 1. Introduction

As a significant engineering material, aluminum alloys are commonly used as materials for everyday applications and industry, such as in rail transit, motor transport and aerospace and shipbuilding industries, owing to its outstanding mechanical properties, exceptional forming performance, excellent corrosion resistance and easy recycling [1,2,3]. The 6082 aluminum alloy, a type of Al-Mg-Si alloy, can be modified via solution and aging treatment. It has outstanding mechanical properties and excellent performance in forming ability [4,5], making it especially suitable for manufacturing products such as bumpers, luggage racks, wing ribs and other lightweight components. Moreover, being a type of metal material, aluminum alloys have good formability due to their face cubic center structure [6,7]. In industrial production, hot processing is commonly employed, including hot extrusion, hot rolling and hot forging. It has been reported that Al-Mg-Si alloys processed via hot extrusion and aging treatment can efficiently absorb energy during crashes. At the same time, they are lightweight [8]. In the modern vehicle industry, Al-Mg-Si alloy blanks are usually chosen for producing outer skin components. Blanks processed via hot rolling display excellent mechanical properties [9]. Producing alloy structural components via plastic deformation is one of the most efficient manufacturing methods in the industry. 

It has been reported that aluminum alloys processed via plastic deformation display a great anisotropy of mechanical properties. Krishna et al. [10] investigated a cold-rolled Al-Mg-Si alloy with different angles along the rolling direction. The flow stress and elongation of the alloy specimens exhibited significant anisotropy. Debih et al. [11] studied the thermodynamic behavior of the 6000 series alloy. The results implied that it exhibits different strains, hardening ability and ductility in the short transverse, long transverse and longitudinal rolling directions. Chen et al. [12] tested the AA6082-T6 alloy with a non-recrystallized structure. They reported that the extruded alloy displays obvious anisotropy of flow stress; namely, the sample with a 45° angle toward the extrusion direction had the lowest strength. The strength of the 0° sample was the highest and the strength of the 90° sample was slightly lower compared with the 0° sample. During the extrusion process of this alloy, its microstructure exhibited a preferred orientation, which has a significant impact on the anisotropic deformation behavior of the material. Chen et al. [13] concluded that the anisotropic mechanical response of the aluminum alloy is affected by a multitude of factors. The characteristics of grain shape, texture, recrystallization and precipitate vary across samples with different directions. Zhou et al. [14] explored the mechanical response of a rolled Al sheet. The rolling deformation led to obvious anisotropic mechanical behaviors, and these phenomena became weaker when the elongated grains transited to equiaxed structures. Vivek et al. [15] claimed that during plastic deformation, both the strain path and loading direction are determined according to the microstructure evolution and texture development of the AA-6061 sheet, which is significant for the formability and results within the evident planar anisotropy. Li et al. [16] tested a Al-Zn-Mg-Sc-Zr alloy sheet and calculated the Schmid factor of the grains. It was obvious that the Schmid factor values of samples with different directions had large differences. The Schmid factors of the 0° and 90° samples were relatively smaller, and their corresponding yield strength was much higher. Huang et al. [17] observed the flake-like precipitates in an Al-Cu-Mg-Mn-Si alloy. They found that the precipitates mainly lay on the {100} planes, which indicated a remarkable orientation. The strengthening mechanism of the precipitates and its effects on the anisotropic mechanical behavior were discussed. In total, the microstructure, including the shape and size of the grain, the type and tensity of the texture, and the type and dimension of the precipitate were all related to the mechanical properties and the anisotropy.

In the industry, most aluminum alloy products are processed at elevated temperatures or serviced in a high-temperature environment, as temperature plays an important role in microstructure evolution. Deng et al. [18] found that a great number of dynamic recrystallization grains are generated during deformation. Alongside that, the dissolution of second-phase particles was observed. Zhao et al. [19] demonstrated that cellular substructures are formed under high-temperature deformation, while Brass {011}<211> and Goss {011}<001> textures with a high intensity are observed during the hot deformation process. Lin et al. [20] studied the hot deformation behavior of a 6082 alloy. They found that the compression temperature ranged from 350 to 500 °C, the strain rate was between 0.01 and 5 s^−1^, a great number of sub-grains were found within the original grains, the sub-grains became coarse due to the migration of the grain boundary, and the orientation difference underwent great changes during the forming process. Furthermore, the final performance of the product is affected by the hot deformation parameters during the forming and processing. High-temperature conditions can induce significant changes in the microstructure, which affects the anisotropy of mechanical properties. Therefore, further research of the anisotropic mechanical behavior and deformation mechanism underlying alloys at elevated temperatures is needed.

With the rapid development of lightweight components, the usage of aluminum alloys has significantly increased [21], including in aviation engine blades, compressor impellers, rocket boosters, automotive engine cylinders and pistons [22,23,24]. These aluminum alloy components operate under extreme conditions, usually in high-temperature environments ranging from 200 to 400 °C [25,26]. During these processes, the texture, grain, precipitate and dislocation of the alloy may undergo a great evolution. Accordingly, the strength and toughness of the components undergo a significant change [27,28]. Therefore, considering the service safety and application promotion of aluminum alloy components, it is necessary to pay attention to the mechanical response of aluminum alloy materials under high-temperature conditions, improve the microstructure and hence their mechanical properties. By now, a certain amount of research has been conducted to explore the mechanical response and microstructure evolution of a 6082 aluminum alloy; however, the deformation conditions were focused on room temperature and compression [29,30,31]. In this study, a 6082-T6 alloy sheet was selected, and the specimens with 0°, 45° and 90° directions along the rolling direction were prepared. The effect of temperature on the tensile behaviors of a 6082-T6 alloy was studied and the related microstructure evolution is discussed in detail. 

## 2. Experimental Procedure

### 2.1. Experimental Material

The experimental material was a 3 mm 6082-T6 alloy sheet, which was used to conduct high-temperature tensile tests. The chemical composition is shown in Table 1.

**Figure 1:**
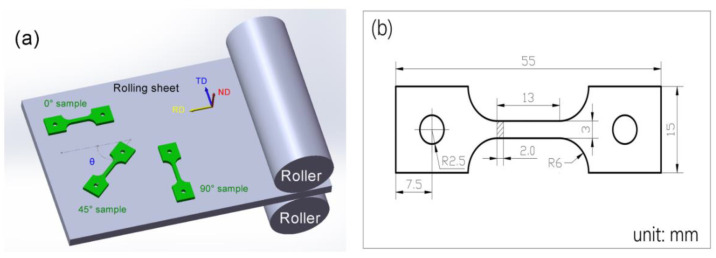
(**a**) Sampling location; and (**b**) the shape and size of the samples (unit: mm).

The tensile samples were machined from the original sheet via wire electrical discharge machining, as shown in Figure 1a. The 0° (rolling direction), 45° (with 45° along the RD) and 90° samples (with 90° along the RD) were machined to explore the anisotropic mechanical response of the 6082-T6 sheet. The size of the samples for high-temperature tension testing are displayed in Figure 1b.

### 2.2. Mechanical Property Test

**Figure 2:**
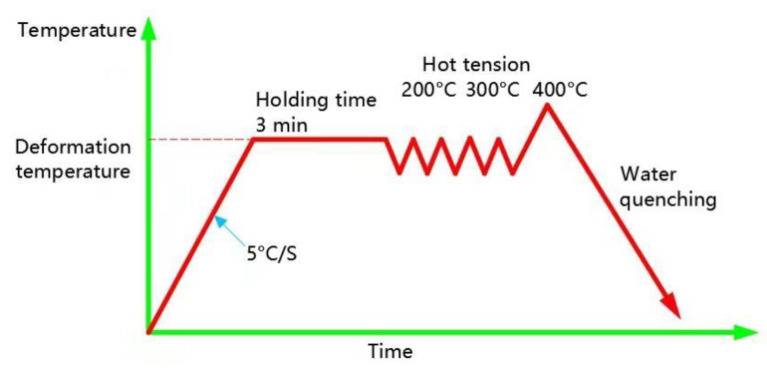
High-temperature tensile testing steps.

The high-temperature tension testing was conducted in an Gleeble 3500 thermal simulation machine (DSI, St. Paul, MN, USA); the tensile strain rate was 0.001 s^−1^. Three samples for each tensile condition were performed to ensure the reliability of the data. Prior to the high-temperature tension testing, the specimens were heated to the target temperature (200, 300 and 400 °C), with a heating rate of 5 °C/s and the samples were held for 3 min once the target temperature was obtained for the purpose of uniform temperature distribution. The load–displacement curves were directly gathered by the sensors. Then, the data were converted into engineering stress–engineering strain curves. The high-temperature tensile testing steps are shown in Figure 2.

### 2.3. Characterization

**Figure 3:**
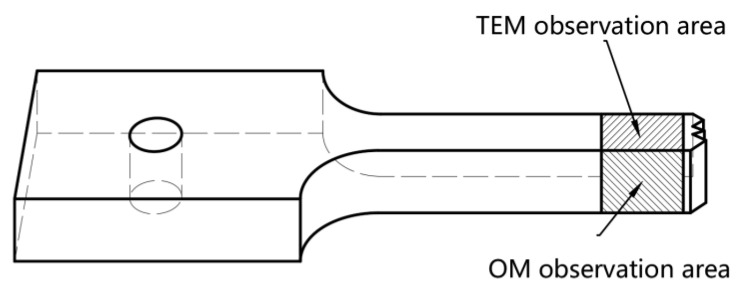
The characterization area of the specimen.

The optical microscopy (AX10, Zeiss, Oberkochen, Germany) samples were cut from the fracture area, as shown in Figure 3. The observation surface was ground and polished, and then the mirror surface was anodized in a solution with 3% HBF_4_ and 97% purified water. The electrolytic voltage was 20 V, the electrolytic time ranged from 1 to 3 min, and a polarization microscope was utilized to observe the microstructure. The fracture morphology observation was conducted via scanning electron microscopy (FEI, Hillsboro, OR, USA), and the fracture surface cut from the failed samples was cleaned with alcohol before the test. As shown in Figure 3, the transmission electron microscopy (TEM) characterization was carried out through Talos F200X (FEI, Hillsboro, OR, USA), and the operation voltage was 300 kV. The TEM observation samples were cut from the fracture zone and ground to a 70 µm thickness. Then, twin-jet thinning was applied to obtain an observation area; the twin-jet thinning voltage was 25 V, the solution consisted of 30% nitric acid and 70% methanol, and the temperature was below −25 °C. An X-ray diffractometer (BRUKER, Billerica, MA, USA) was used to measure the macro-texture of the original sample; the TD–RD plane of the sample was ground and polished, and the pole figures of (111), (200), (220) and (311) were tested. 

## 3. Experimental Results

### 3.1. High-Temperature Flow Behavior

**Figure 4:**
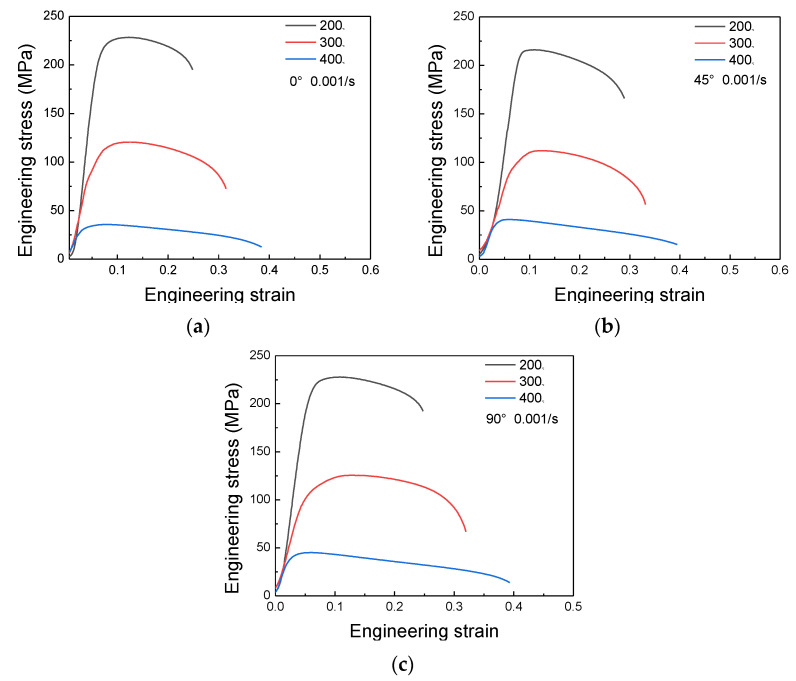
The true stress–strain curves of a rolled 6082-T6 aluminum alloy under different tensile temperatures: (**a**) 0°; (**b**) 45°; and (**c**) 90°.

Figure 4 displays the engineering stress–engineering strain data for the 6082-T6 alloys, when the tensile strain rate was 0.001 s^−1^ and the temperatures were 200, 300 and 400 ℃. At the initial stage of high-temperature tension, the alloy underwent elastic deformation and the stress increased up rapidly. After reaching the yield point, the rising trend slowed down and the stress level of the material was the competitive relationship between work hardening and dynamic softening. The work hardening is typically attributed to the dominating strain hardening effect caused by dislocation multiplication. It is worth noting that the peak stress was obtained at a lower strain when deformed at a higher temperature. This can explained by the more intense dynamic softening of the alloy during higher-temperature deformation. As shown in Figure 4a, at a temperature of 200 °C, the peak stress of the 0° specimen was 228 MPa; the corresponding strain was 0.08. At a temperature of 400 °C, the peak stress was 31 MPa and the corresponding strain fell to 0.02. As reported, the softening effects include various types of dynamic recovery (DRV), dynamic recrystallization (DRX), grain coarsening, particle growth, etc. [32,33,34,35]. When the stress reaches the peak point, the work hardening effects and softening effects are in balance, and the stress remains constant. With increasing strain, the softening effects take the lead, and the stress begins to decline slowly. At the last stage of high-temperature tension, the cavities begin to merge due to the triaxial effect, leading to the local necking, which facilitates the failure of the sample. Similar stress–strain curve trends have been reported by Vilamosa et al. [36] for an Al-Mg-Si alloy, Chen et al. [37] for a 6061-T6 alloy, Guo et al. [38] for a 2124-T4 alloy and Xiao et al. [39] for an AA7075 alloy. In total, during the high-temperature deformation, the stress–strain curves are a result of the strengthening and softening effects.

For a deeper analysis of the anisotropic mechanical properties, the max stresses of different tensile conditions were explored. In previous studies, Guo et al. [40] found that a hot-rolled Al-Zn-Mg-Cu alloy exhibits obvious mechanical anisotropy at low-temperature tension. Ye et al. [41] reported that the anisotropy becomes weaker with increasing temperature. The present research was consistent with previously reported results. At a temperature of 200 °C, the max stress for the rolling direction samples was the highest. The value of the 90° sample was comparable to the 0° sample. The stress of the 45° sample remained at the lowest level; the stress was 230, 217 and 229 MPa for the 0°, 45° and 90° samples, respectively. This indicates that a 6082-T6 alloy exhibits evident mechanical property anisotropy. The grain shape and macro texture were the main factors causing the mechanical anisotropy [42,43]. When the temperature was raised to 300 ℃, the stresses of the 0° and 90° samples were comparable, with the specific values being 121 and 120 MPa. The stress of the 45° sample was 112 MPa, which is relatively lower. It was concluded that the differences in the max stress among the samples with different directions became less evident. At 400 ℃, the stresses of the three different samples were around 40 MPa. There was no obvious difference in the stress levels. In elevated-temperature deformation, the continuously stored deformation and thermal energies inside the specimens promoted dynamic recovery and recrystallization, which resulted in a decline in dislocation density. The slip was easier to activate; meanwhile, the grain shape and original texture underwent a great change. All these factors may be attributed to the weakening of anisotropy.

### 3.2. Fracture Behavior

**Figure 5:**
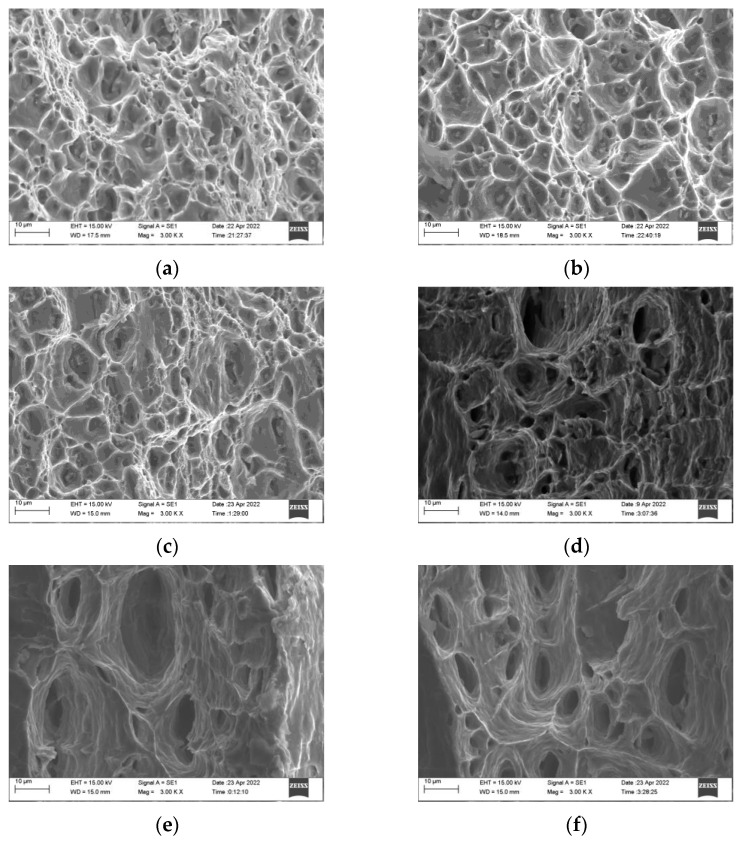
Tensile fracture morphology under different deformation conditions: (**a**) 200 °C, 0°; (**b**) 200 °C, 45°; (**c**) 200 °C, 90°; (**d**) 400 °C, 0°; (**e**) 400 °C, 45°; and (**f**) 400 °C, 90°.

Figure 5a–f shows the fracture surfaces of the rolled 6082-T6 alloy according to the cutting direction relative to the rolling direction. All the samples displayed ductile fracture behavior, regardless of the direction. This is in accordance with previous studies, among which Kashyap et al. [44] found that the deformation of an ultrafine-grained Al-Mg-Si alloy tensile sample becomes more homogeneous with increasing temperature. Khan et al. [45] tested the 7075 aluminum alloy at 20–420 °C. The number of dimples increased with increasing temperature and ductile fractures were the dominant failure mode. He et al. [46] explored the hot tensile behavior of a 7046 aluminum alloy, where a great number of dimples were detected and ductile fracture characteristics were observed. The tensile fractures deformed at 200 °C are displayed in Figure 5a–c, where can be observed that there are some cleavage surfaces and a large number of dimples, and the dimples are shallow and have an uneven size distribution, with both small equiaxed dimples and larger oval dimples, hence exhibiting typical ductile fracture characteristics. At 400 °C, the cleavage surface disappeared, the dimples were deeper and larger, and the shape and size tended to be uniform, exhibiting better ductility. From the fracture surface, as the temperature increased, the ductility of the alloy gradually increased. The main reason is that, as the recovery and recrystallization progress, the coordinated deformation ability of the grains was enhanced, thereby improving the plasticity of the alloy. This is consistent with the mechanical property results. 

## 4. Discussion

**Figure 6:**
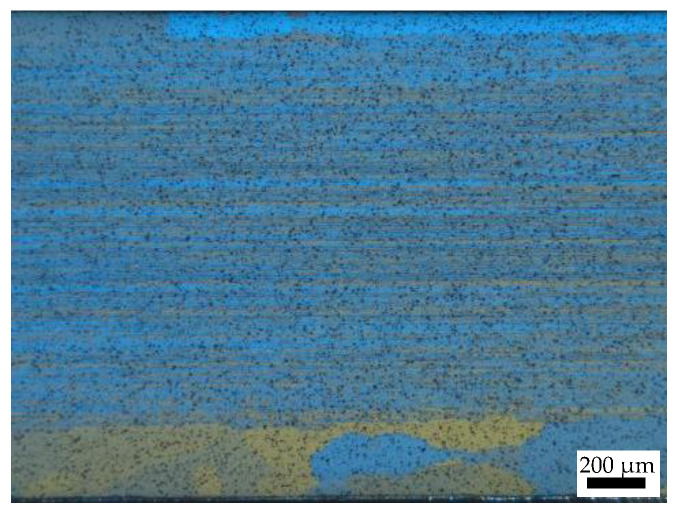
OM images of the grain structure of the original materials.

The phenomenon of anisotropy of an aluminum alloy depends on its grain morphology [13,40]. From Figure 6, it can be seen that the effect of rolling force significantly elongated the grains along the main deformation direction. The alloy consisted of fiber grains with well-defined grain boundaries. The width of the grains was 20–50 μm and some equiaxed recrystallized grains were visible near the grain boundaries. The coarse particles were dissolved into the Al matrix during the hot rolling. There was no thick dendritic and net-like second phase [47], implying that a homogeneous microstructure was achieved. Previous studies showed that elongated grains can exacerbate the anisotropic mechanical properties of the material [48,49]. Yang et al. [50] reported that the elongated grain microstructure of a 7075 aluminum alloy leads to anisotropic flow stress. The strengths of the 0° specimens were the highest in the temperature range of 320–340 °C. Ye et al. [51] found that the fiber grains of a 6063-T4 aluminum alloy contributes to the highest flow stresses of the 0° specimens. The elongated grains resulted in different densities of grain boundaries in different directions. The grain boundaries hindered the movement of the dislocations, causing a large amount of dislocations to accumulate at the grain boundaries. These dislocations cause lattice distortion within the grain boundaries, thereby the strengthening effect varies with different grain boundary densities [52]. The density of the grain boundary in the 0° sample was significantly higher than those in the 45° and 90° samples, resulting in a higher material strength. However, the accumulation of the dislocations cannot relax the stress concentration caused by the dislocations, making the material more prone to fracture under external forces and resulting in poor plasticity in the 0° direction.

The microstructure of an aluminum alloy after rolling deformation usually exhibits an obvious preferred orientation, which is closely related to its mechanical property anisotropy [53]. The SFs and orientation distribution functions (ODFs) are used to describe the anisotropy [50,54]. The expression of the SFs can be written as follows:

(1) $$\tau =\sigma_{y}=\frac{F}{A}\cos\varphi \cos\lambda$$

(2) M=cosφcosλ

where *τ* is the critical resolved shear stress, *A* is the loaded section, *φ* is the angle of the normal to the slip plane and loading axis, *λ* is the angle between the loading axis to the slip direction, *σ_y_* is the yield stress and *M* is the Schmid factor. The relationship shown in Equations (1) and (2) has been confirmed in previous studies [40,55]. The results show that the 0° specimens have the lowest Schmid factor and exhibit the highest yield strength. The higher Schmid factor value of the 45° sample was one of the reasons for the relatively lower strength. Therefore, the primary cause of the results presented in Figure 4 can also be deduced to be the same reason as that for the 6082-T6 alloy deformation at 200 °C.

**Figure 7:**
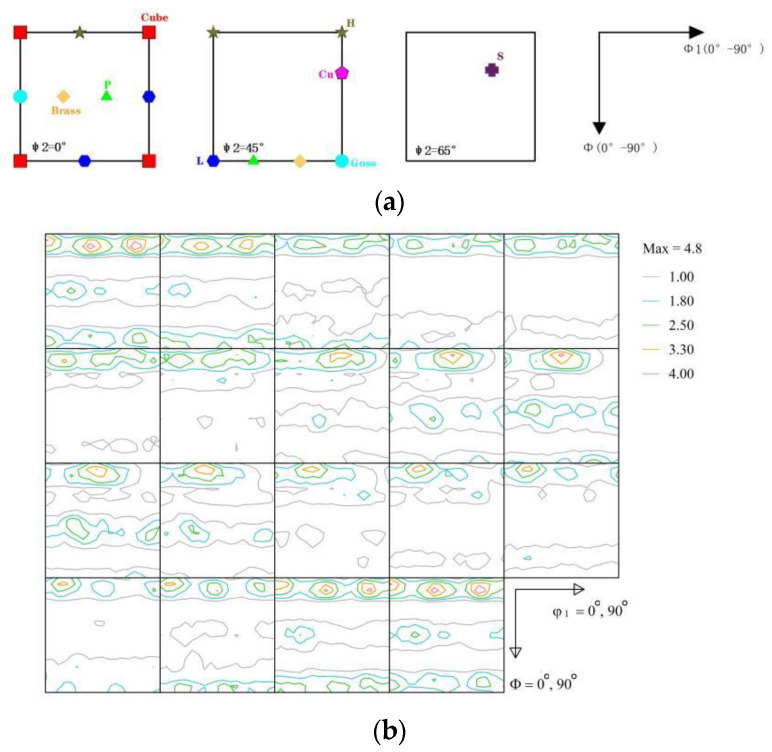
(**a**) Main texture components in the ODF sections (φ_2_=0°, φ_2_=45°, φ_2_=65°); and (**b**) the ODF map of the 6082-T6 sheet.

Figure 7a shows the main texture components of the face-centered cubic metal. Figure 7b displays the ODF constant of the 6082-T6 aluminum alloy rolled sheet. The overall texture of the rolled sheet was the cube {011}<100> and brass components {110}<112>. The cube component {011}<100> has a typical recrystallization texture. It was reported that the cube component has little effect on the anisotropy of mechanical properties. The brass component {110}<112> was developed due to the prior rolling deformation process, as it could enhance the strength in the rolling direction and weaken the strength of the sample with a 45° along rolling direction, which resulted in the anisotropic mechanical properties. Zhou et al. [14] explored 2024 aluminum alloy sheets and claimed that the transformation from the deformed into the recrystallized texture was responsible for the reduction in anisotropy. Li et al. [16] studied the anisotropic mechanical properties of an Al-Zn-Mg-Sc-Zr sheet and concluded that the brass component {110}<112> was the main reason for the anisotropy. Therefore, the brass component was another reason that led to the anisotropic mechanical properties. 

**Figure 8:**
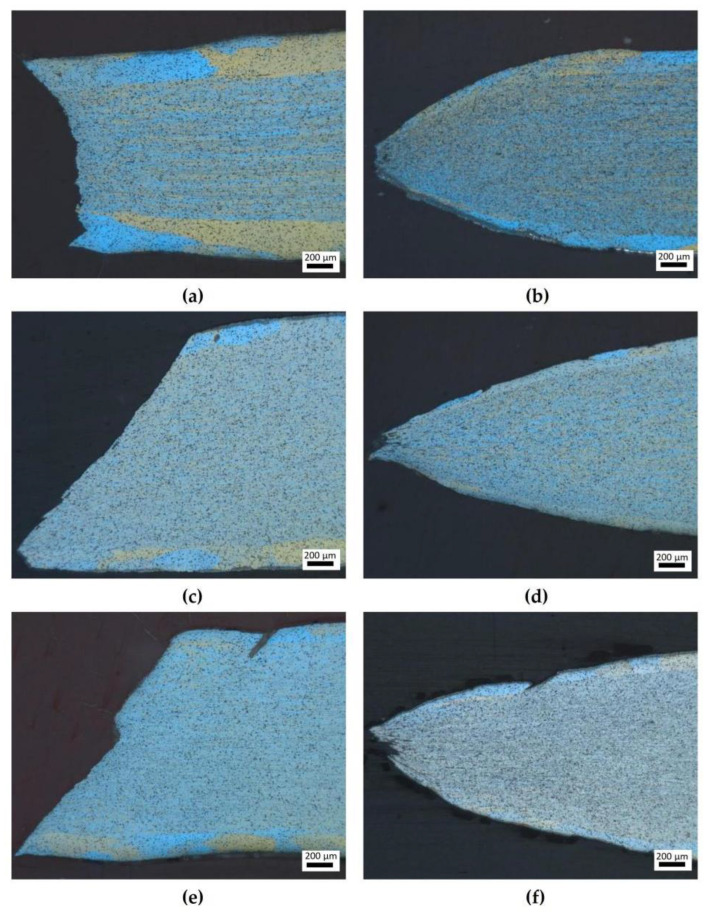
OM images of the microstructure after deformation: (a) 0°, 200 °C; (**b**) 0°, 400 °C; (**c**) 45°, 200 °C; (**d**) 45°, 400 °C; (**e**) 90°, 200 °C; and (**f**) 90°, 400 °C.

Compared with Figure 8a,b, in Figure 8c–f, the tensile deformation temperature increased from 200 °C to 400 °C, respectively, and the elongation of the 6082-T6 aluminum alloy also increased. This is because high temperatures can improve the diffusion ability of atoms, significantly enhance the fluidity of the alloy, and enhance the deformation ability of the aluminum alloy. The grains near the fracture surface underwent shear deformation along the 45° direction. On the other hand, some fine grains were observed in the deformation at 400 °C. High temperatures and large plastic deformations can provide more stored energy, which plays a role in triggering dynamic recrystallization. It is widely accepted that dynamic recrystallization facilitates the plastic deformation ability of metal materials and is useful for inducing uniform deformation. Moreover, it can alleviate the anisotropic mechanical properties as the deforming texture transits into the recrystallized texture. From Figure 8a–f, it can be seen that during the tensile process, fractures occurred along the 45° tensile direction due to the shear stress. When the deformation reached a certain degree, the internal micropores of the material nucleated and grew, and the adjacent pores began to aggregate, ultimately leading to the fracture of the tensile sample. A certain number of pores were formed near the fracture surface, as shown in Figure 8b,d,f. 

**Figure 9:**
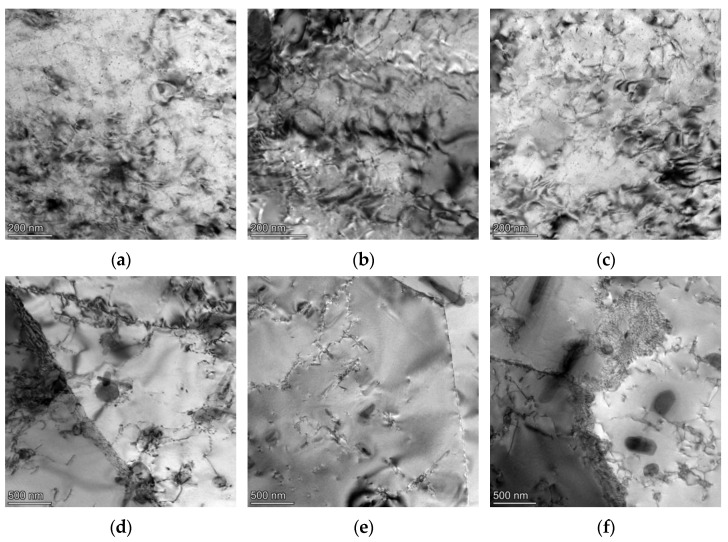
TEM morphology of a 6082-T6 alloy after tension under different conditions: (**a**) 200 °C, 0°; (**b**) 200 °C, 45°; (**c**) 200 °C, 90°; (**d**) 400 °C, 0°; (**e**) 400 °C, 45°; and (**f**) 400 °C, 90°.

Figure 9 shows TEM photos of a 6082-T6 aluminum alloy sheet in the 0°, 45° and 90° directions under different deformation temperatures. During the tensile deformation process at 200 °C, the matrix exhibited a high-density dislocation structure and polygonal dislocation cells, resulting in characteristics of the restored microstructure. At lower deformation temperatures, recovery dominated and the dislocation slip canceled out the heterologous dislocations, causing dislocation entanglement to rearrange and form a substructure; the strength of the alloy began to decrease. At 400 °C, the dislocation density further decreased. When the deformation temperature was elevated, the energy storage in the alloy gradually accumulated, inducing a continuous absorption of dislocations at the grain boundary. Meanwhile the sub-grains began to merge and grow to achieve dynamic recrystallization, leading to a significant decline in the dislocation density within the grain. The typical recrystallization grain characteristics, namely triangular grain boundaries, appeared in the alloy. It is worth noting that after tension at 400 °C, coarse precipitates began to appear inside the grains and along the grain boundaries, which weakened the effect of precipitation strengthening [56]. Bembalge et al. [57] characterized the tensile specimen of a 6063 alloy after deformation at 350 ℃. Reductions in the dislocation and formation of the recrystallized grains were observed. Ye et al. [41] observed the coarsening of precipitates of a 7075 aluminum alloy after deformation at an elevated temperature, which resulted in a decrease in mechanical properties. Both the dynamic recrystallization and precipitate coarsening contributed to the decreased flow stress [58,59].

## 5. Conclusions

In this study, specimens with different angles (0°, 45°, 90°) along a rolled 6082-T6 aluminum alloy sheet were prepared. High-temperature tensile tests were carried out to investigate the influence of temperature on mechanical properties. The tensile temperatures were 200 °C, 300 °C and 400 °C, and the tensile strain rate was 0.001 s^−1^.

(1) The engineering stress of the 6082-T6 aluminum alloy increased rapidly with the increase in engineering strain. After the yield point, the rising speed slowed down and reached the peak. Then, the stress began to decline before the fracture. The tensile process of the alloy was an interaction of work hardening and dynamic softening.
(2) The stress level of all the samples decreased with increasing temperature. This can be explained by dynamic recovery, dynamic recrystallization and precipitate coarsening. The specimens displayed ductile fractures, and the dimples became deeper and larger at higher tensile temperatures.
(3) When deformed at 200 °C, the alloy exhibited obvious anisotropy. This can be attributed to the original elongated grains. Alongside that, the brass component also resulted in the anisotropy. With increasing temperature. The anisotropy weakened at a temperature of 400 °C and there was no evident anisotropy because it was easier for the slip to be activated at an elevated temperature, and a great amount of deformation structure in the original material turned into a recrystallized structure.

## Figures and Tables

**Table 1:** Chemical composition of a 6082-T6 aluminum alloy sheet.

Element	Si	Fe	Cu	Mn	Mg	Cr	Zn	Ti	Al
wt%	0.89	0.2	0.1	0.43	0.75	0.1	0.02	0.09	Balance

## Data Availability

Not applicable.
